# *RUNX1-205*, a novel splice variant of the human *RUNX1* gene, has blockage effect on mesoderm–hemogenesis transition and promotion effect during the late stage of hematopoiesis

**DOI:** 10.1093/jmcb/mjaa019

**Published:** 2020-04-21

**Authors:** Wencui Sun, Jiahui Zeng, Jing Chang, Yuan Xue, Yonggang Zhang, Xu Pan, Ya Zhou, Mowen Lai, Guohui Bian, Qiongxiu Zhou, Jiaxing Liu, Bo Chen, Feng Ma

**Affiliations:** 1 Institute of Blood Transfusion, Chinese Academy of Medical Sciences & Peking Union Medical College (CAMS & PUMC), Chengdu 610052, China; 2 State Key Laboratory of Biotherapy, Sichuan University, Chengdu 61006, China; 3 State Key Laboratory of Experimental Hematology, CAMS & PUMC, Tianjin 300020, China

**Keywords:** mesoderm–hemogenesis transition, hematopoiesis, hematopoietic stem/progenitor cells (HSPCs), *RUNX1*, mesoderm, embryonic stem cells (ESCs)

## Abstract

Runt-related transcription factor 1 (*RUNX1*) is required for definitive hematopoiesis; however, the functions of most human *RUNX1* isoforms are unclear. In particular, the effects of *RUNX1-205* (a novel splice variant that lacks exon 6 in comparison with *RUNX1b*) on human hematopoiesis are not clear. In this study, a human embryonic stem cell (hESC) line with inducible *RUNX1-205* overexpression was established. Analyses of these cells revealed that induction of *RUNX1-205* overexpression at early stage did not influence the induction of mesoderm but blocked the emergence of CD34^+^ cells, and the production of hematopoietic stem/progenitor cells was significantly reduced. In addition, the expression of hematopoiesis-related factors was downregulated. However, these effects were abolished when *RUNX1-205* overexpression was induced after Day 6 in co-cultures of hESCs and AGM-S3 cells, indicating that the inhibitory effect occurred prior to generation of hemogenic endothelial cells, while the promotive effect could be observed during the late stage of hematopoiesis. This is very similar to that of *RUNX1b*. Interestingly, the mRNA expression profile of *RUNX1-205* during hematopoiesis was distinct from that of *RUNX1b*, and the protein stability of RUNX1-205 was much higher than that of RUNX1b. Thus, the function of *RUNX1*-*205* in normal and diseased models should be further explored.

## Introduction

Runt-related transcription factor 1 (*RUNX1*) is the key gene for human hematopoiesis, which plays a critical role in the development of hemogenic endothelium and hematopoietic stem cell (HSC) formation during embryogenesis (Chen et al., 2009). Embryonic stem cells of Runx1 knockout mouse are unable to undergo hematopoietic differentiation, which can be rescued by Runx1 re-expression (Nishimura et al., 2004).

*RUNX1* gene is 216 kb long and located on human chromosome 21 (21q22.12) and has two promoters termed P1 (distal) and P2 (proximal). Seventeen transcripts of human *RUNX1* have been identified, among which *RUNX1a/b/c* have been well studied (Shinobu et al., 2007; Challen and Goodell, 2010; Shinobu et al., 2012; Draper et al., 2016). Their expressions are regulated by P1 and P2 promoters with alternative splicing, respectively (Levanon et al., 2001). *RUNX1b/c* have the same DNA-binding region and transcriptional regulatory domains while a few differences at the amino terminus, which indicated similar functions. *RUNX1a* is regulated by the P2 promoter, just like *RUNX1b*, but lacks the transcriptional regulatory domains and has an antagonism against the *RUNX1b/c*. The *RUNX1a* could stimulate the hematopoiesis while *RUNX1b/c* show a repressor for hematopoiesis at the earliest stage (Tsuzuki et al., 2007; Ran et al., 2013; Chen et al., 2017). These *RUNX1* varieties exhibit distinct expression patterns during hematopoiesis (Challen and Goodell, 2010).

*RUNX1-205*, a novel splice variant of human *RUNX1*, is the sole and longest protein encoded by the complete coding region other than *RUNX1a/b/c. RUNX1-205* lacks exon 6 in comparison with *RUNX1b* due to alternative splicing and plays a key role in ovarian cancer (Nanjundan et al., 2007; Hong and Fritz, 2019); however, its function in human hematopoiesis is unclear, which might be the final blank field of human *RUNX1* variants research. The homologous mouse gene has complex functions in hematopoiesis (Komeno et al., 2014), indicating that *RUNX1-205* plays an important role in human hematopoiesis. However, the function of *RUNX1-205* in human hematopoiesis has not been explored using an *in vitro* system.

Although *RUNX1-205* lacks exon 6, it retains an intact Runt-related DNA-binding domain and is highly homologous to *RUNX1b*. At the stage of mesoderm−hemogenesis transition, overexpression of *RUNX1b* at early stage blocks human hematopoiesis (Chen et al., 2017). The mRNA expression profile of *RUNX1-205* and its functional similarities and differences with *RUNX1b* during hematopoiesis need to be elucidated. The role of *RUNX1-205* in human physiology and pathologies must be explored further.

## Results

### Genome structure analysis and alignment of RUNX1 homologous genes

The human *RUNX1* gene has 12 exons, which encompass a runt homology domain (exons 3–5), an mSin3A interaction domain (exon 6), and a transactivation domain (exons 7B and 8). Exon 6 is deleted in human *RUNX1-205* in comparison with *RUNX1b* (Supplementary Figure S1A). The function of *RUNX1-205* is poorly understood. A splice variant in mouse called *Runx1-202* has been reported, which is highly homologous to human *RUNX1-205* (Komeno et al., 2014; Supplementary Figure S1B). A BLAST search of higher vertebrates from fish to human revealed that splice variants homologous to *RUNX1-205* have been highly conserved during evolution (Supplementary Figure S1C).

### RUNX1-205/hESCs exhibit inducible RUNX1-205 overexpression and normal pluripotency potential

**Figure 1:**
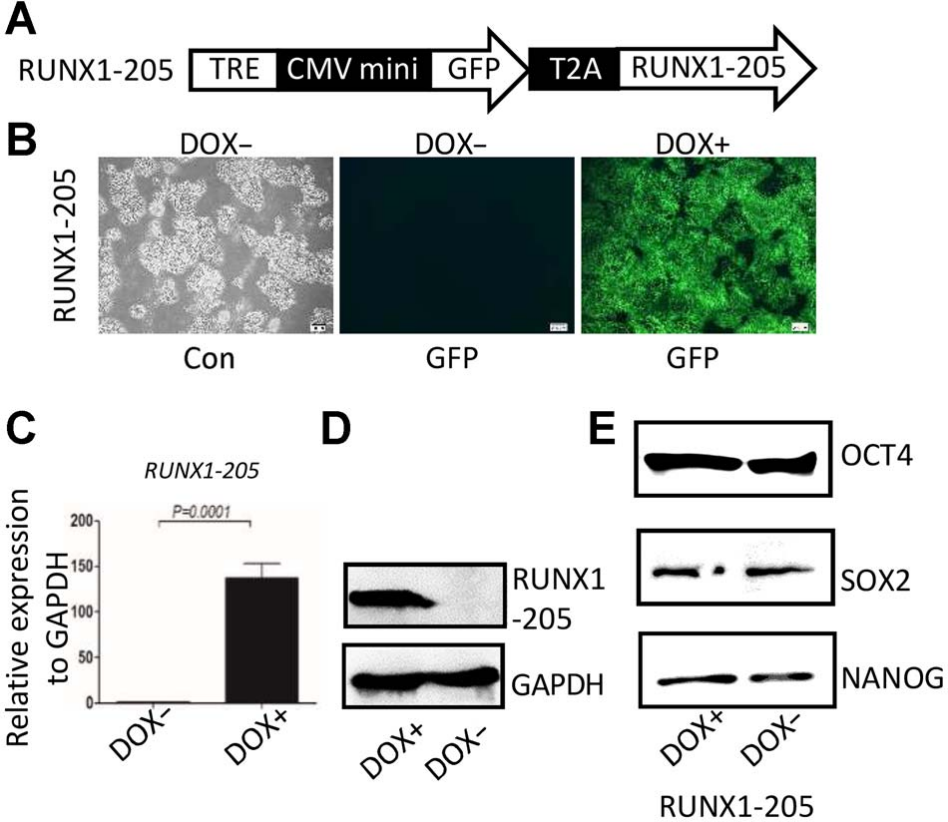
Inducible expression of *RUNX1b* in hESCs (H1) and detection of potentials. (**A**) Schematic representation of the *piggyBac* construct harboring *RUNX1-205*. TRE, Tet-on regulation element; CMV mini, cytomegalovirus minimum promoter; T2A, *Thosea asigna* virus 2A peptide. (**B**) Fluorescence and phase contrast images of *RUNX1-205*/hESCs treated with or without DOX. (**C**) Ectopic expression of *RUNX1-205* detected by qRT-PCR. *GAPDH* was used as an internal control. *P* < 0.05 was considered significant. (**D**) Ectopic expression of RUNX1-205 detected by western blotting. GAPDH was used as a loading control. (**E**) Expression of stemness-specific markers (OCT4, SOX2, and NANOG) in *RUNX1-205*/hESCs with or without DOX treatment was detected by western blotting. GAPDH was used as a loading control.

*RUNX1-205*/hESCs were established as described previously (Chen et al., 2017; Figure 1A) and treated with doxycycline (DOX) for 48 h. Fluorescence microscopy, quantitative reverse transcription PCR (qRT-PCR), and western blot analyses demonstrated that *RUNX1-205* overexpression was efficiently induced (Figure 1B–D). Western blot analysis also demonstrated that stemness-specific markers, including OCT4, SOX2, and NANOG, were normally expressed in *RUNX1-205*/hESCs (Figure 1E), demonstrating that these cells have a normal pluripotency potential.

### Induction of RUNX1-205 overexpression at early stage blocks human hematopoiesis, whereas the induction at late stage promotes hematopoiesis

**Figure 2:**
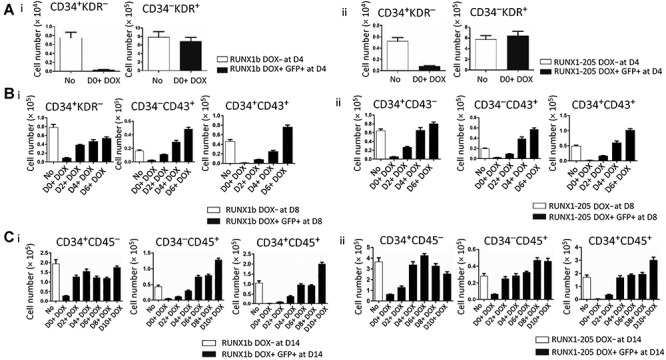
Ectopic expression of *RUNX1b* and *RUNX1-205* blocks hematopoiesis in co-cultures with AGM-S3 cells. Co-cultured *RUNX1b*/hESCs or *RUNX1-205*/hESCs were treated without (No) or with DOX from D0, D2, D4, D6, D8, or D10 and analyzed by FACS at D4, D8, or D14 using antibodies against CD34/KDR, CD34/CD43, or CD34/CD45. The CD34^+^KDR^−^ and CD34^−^KDR^+^ populations at D4 (**A**), CD34^+^CD43^−^, CD34^−^CD43^+^, and CD34^+^CD43^+^ populations at D8 (**B**), and CD34^+^CD45^−^, CD34^−^CD45^+^, and CD34^+^CD45^+^ populations at D14 (**C**) were compared between noninduced co-cultures and the GFP^+^ fraction of co-cultures treated with DOX.

The effects of *RUNX1-205* overexpression on human hematopoiesis differed according to when DOX treatment was initiated. Fluorescence activated cell sorting (FACS) analysis of co-cultured *RUNX1-205*/hESCs and the aorta-gonad-mesonephros (AGM-S3) cells revealed that treatment with DOX from Day 0 (D0) did not influence mesoderm induction (measured by product of KDR^+^CD34^−^; Figure 2A; Supplementary Figure S3A), but severely blocked early hematopoiesis (measured by product of KDR^−^CD34^+^). However, this effect was attenuated or abolished when DOX treatment was initiated after D6, and the hematopoiesis could even be stimulated in some degree with DOX treatment from D10 (Figure 2B and C; Supplementary Figure S3B and C). Generation of CD34^+^ cells was prevented by induction of *RUNX1-205* overexpression at early stage (especially from D0), and consequently, both CD34^+^CD43^+^ and CD34^+^CD45^+^ populations were lost. However, the production of CD34^+^ populations was almost normal when *RUNX1-205* overexpression was induced from D6 or later and even significantly enhanced with *RUNX1-205* induction from D10. This indicates that the development of hemogenic endothelium was blocked by induction of *RUNX1-205* overexpression at early stage but not affected by *RUNX1-205* induction after D6 (Figure 2). These effects of *RUNX1-205* on hematopoiesis are similar to those of *RUNX1b*.

### Induction of RUNX1-205 overexpression at early stage downregulates hematopoiesis-related gene expression in hESCs during embryoid body formation and in co-cultures with AGM-S3 cells

**Figure 3:**
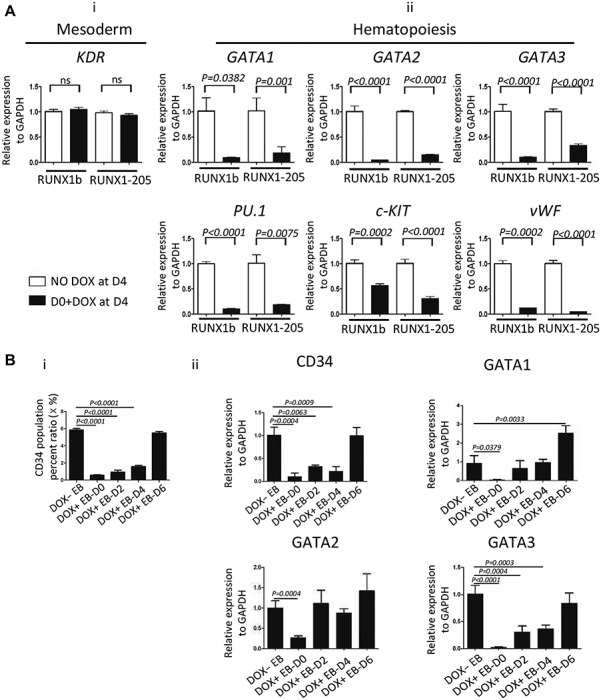
Induction of *RUNX1b* and *RUNX1-205* overexpression at early stage in co-cultures with AGM-S3 cells or during EB formation downregulates hematopoiesis-related genes. (**A**) Co-cultured *RUNX1b*/hESCs or *RUNX1-205*/hESCs were treated without or with DOX from D0 and analyzed by qRT-PCR at D4. Expression of *KDR*, which is related to mesoderm induction, was stable (**i**), while important hematopoiesis-related genes were downregulated (**ii**). (**B**) FACS analysis at EB-D10 showed that overexpression of *RUNX1-205* at early stage of EB formation blocked production of CD34^+^ cells (**i**), while qRT-PCR analysis showed that hematopoietic markers, such as *CD34*, *GATA1*, *GATA2*, and *GATA3*, were downregulated (**ii**). *P* < 0.05 was considered significant.

FACS and qRT-PCR analyses demonstrated that the KDR^+^ population among green fluorescent protein positive (GFP^+^) cells was unaffected at D4 in co-cultures of *RUNX1-205*/hESCs and AGM-S3 cells treated with DOX from D0, indicating that *RUNX1-205* overexpression did not block mesoderm induction (Figures 2A and 3Ai; Supplementary Figure S3A). However, qRT-PCR analysis demonstrated that hematopoiesis-related genes, such as *GATA1*, *GATA2*, *GATA3*, *c-KIT*, *vWF*, and *PU.1*, were downregulated at D4 (Figure 3Aii). During embryoid body (EB) formation by *RUNX1-205*/hESCs, qRT-PCR and FACS analyses at EB-D10 demonstrated that the production of CD34^+^ cells was severely reduced by induction of *RUNX1-205* overexpression at early stage. qRT-PCR analysis also revealed that hematopoiesis-related genes, such as *GATA1*, *GATA2*, and *GATA3*, were downregulated (Figure 3B). In all these experiments, the earlier DOX was added, the inhibitory effect on hematopoiesis was more severe (Figure 3A and B). This indicates that overexpression of *RUNX1-205* at early stage could block hematopoiesis of hESCs in co-cultures with AGM-S3 cells and during EB formation, which is very similar to that of *RUNX1b*.

### Induction of RUNX1-205 overexpression at early stage blocks colony formation, whereas the induction at late stage mildly stimulates hematopoietic potentials

**Figure 4:**
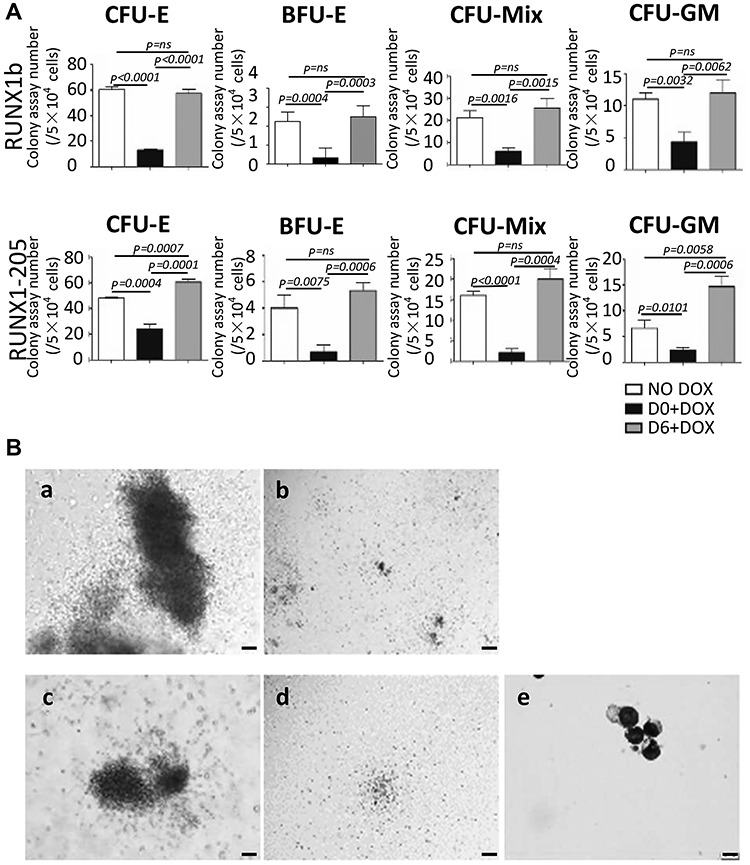
Hematopoietic colony-forming assay. Co-cultured *RUNX1b*/hESCs or *RUNX1-205*/hESCs were treated without or with DOX from D0 or D6 and examined at D14 to determine their hematopoietic potential. (**A**) Numbers of colonies derived from 5 × 10^4^ co-cultured cells. *P* < 0.05 was considered significant. (**B**) Typical morphologies of BFU-E (**a**), CFU-E (**b**), CFU-Mix (**c**), and CFU-GM (**d**) colonies. Scale bar, 100 μm. MGG staining of cells in BFU-E colonies (**e**). Scale bar, 10 μm.

The hematopoietic colony-forming assay was performed with co-cultures of *RUNX1-205*/hESCs and AGM-S3 cells at D14. In comparison with the noninduced control, induction of *RUNX1-205* overexpression from D0 significantly blocked formation of burst-forming unit-erythroid (BFU-E), colony-forming unit-erythroid (CFU-E), colony-forming unit-mixed (CFU-Mix), and colony-forming unit-granulocyte/macrophage (CFU-GM) colonies (*P* < 0.05); however, the colony numbers were normal with *RUNX1-205* induction from D6 (Figure 4A), consistent with the FACS results (Figure 2B and C; Supplementary Figure S3B and C). These results indicate that overexpression of *RUNX1-205* at early stage could block human hematopoiesis, similar to *RUNX1b*. Meanwhile, a mild stimulation effect could be observed on CFU-GM and CFU-E with *RUNX1-205* overexpression at D6, which was not observed statistically for *RUNX1b*. The morphologies of hematopoietic colonies were examined by phase contrast microscopy (Figure 4Ba–d), and erythroid cells were detected by May-Grunwald-Giemsa staining (MGG; Figure 4Be).

### RUNX1-205 overexpression at early stage blocks human mesoderm–hemogenesis transition

**Figure 5:**
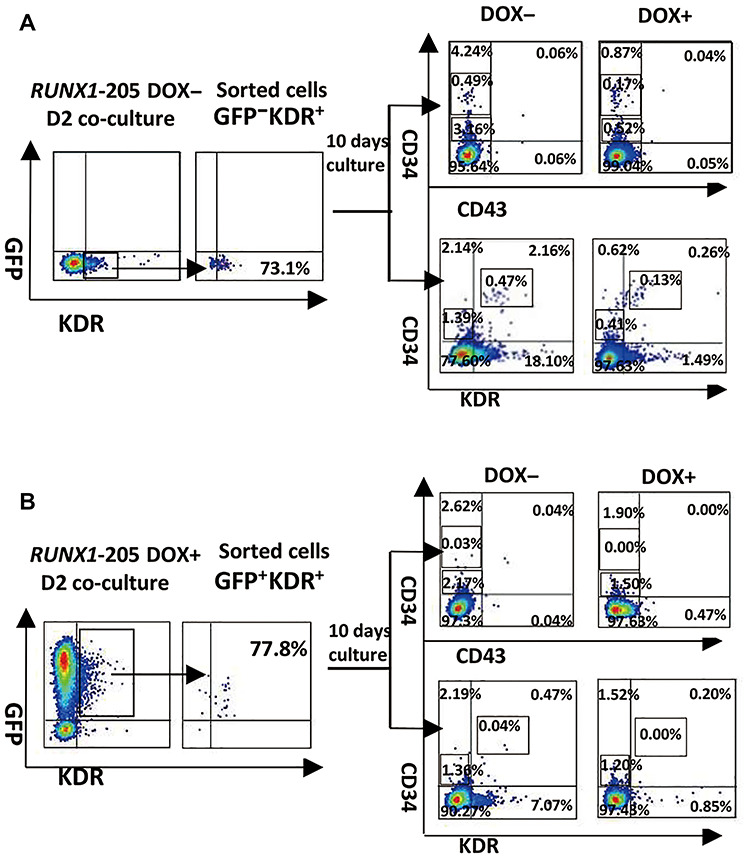
Sorting and hematopoietic potential detection at early stage of co-culture with AGM-S3 cells. KDR^+^ cells were sorted from noninduced or induced *RUNX1-205*/hESC co-cultured with AGMS-3 cells at D2. About 5 × 10^3^ sorted cells were re-plated in the 24-well plate with irradiated AGMS-3 cells, treated with or without DOX, and subjected to FACS analysis at D10 using antibody combinations against CD34/KDR and CD34/CD43.

KDR^+^ cells sorted from *RUNX1-205*/hESC (induced or not) co-cultured with AGMS-3 at D2 were further cultured in irradiated AGMS-3 with or without DOX (named as D++, D+−, D−+, D−−). FACS analysis indicated that the production of CD34^+^CD43^−^, CD34^+^KDR^−^, and CD34^+^KDR^+^ populations significantly dropped after induction of *RUNX1-205* overexpression from D2 (D−+ vs. D−−; Figure 5A). *RUNX1-205* induction from D0 mostly blocked the emergence of CD34^high^CD43^−^ and CD34^high^KDR^+^ populations and completely inhibited these CD34^high^ populations if continuously induced from D0 to D4, but showed milder effect on the production of CD34^low^CD43^−^ and CD34^low^KDR^−^ populations (D+− and D++; Figure 5B). The above results indicated that ectopic expression of *RUNX1-205* at early stage could dampen the development of CD34^+^ endothelium and block mesoderm–hemogenesis transition.

### Induction of RUNX1-205 overexpression does not affect the hematopoiesis stage after mesoderm–hemogenesis transition but even later stage

**Figure 6:**
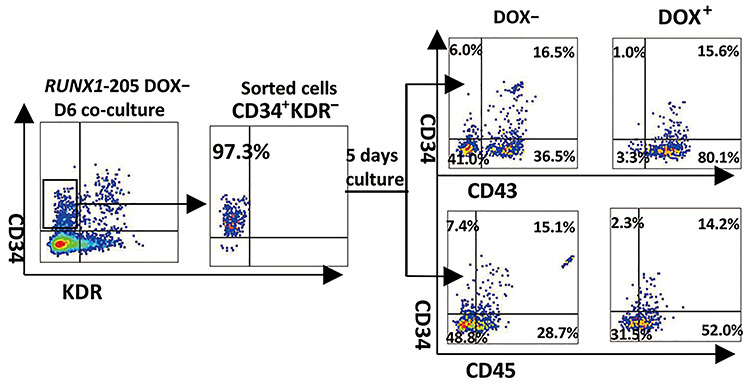
Sorting and hematopoietic potential detection at late stage of co-culture with AGM-S3. CD34^+^KDR^−^ cells were sorted from noninduced *RUNX1-205*/hESC co-cultured with AGMS-3 cells at D6. About 5 × 10^3^ cells were cultured in the 48-well plate containing FLHD medium, treated with or without DOX, and finally analyzed by FACS at D5 using antibody combinations against CD34/CD43 and CD34/CD45. Induction of *RUNX1-205* overexpression at late stage did not influence the production of CD34^+^CD43^+^ and CD34^+^CD45^+^ hematopoiesis stem/progenitor cells (HSPCs), but promoted the production of CD34^−^CD43^+^ and CD34^−^CD45^+^ blood cells.

CD34^+^KDR^−^ populations sorted from *RUNX1-205*/hESCs co-cultured with AGMS-3 at D6 were further cultured for 5 days. FACS results showed that the induction of *RUNX1-205* overexpression at the hematopoiesis stage (D6) did not affect the production of CD34^+^CD43^+^ and CD34^+^CD45^+^ populations, but promoted CD34^−^CD43^+^ and CD34^−^CD45^+^ populations (Figure 6). This indicated that *RUNX1-205* induction after the mesoderm−hemogenesis transition could not influence the development of hematopoiesis stem/progenitor cells (HSPCs) but promoted the production of CD34^−^ blood cells at later stage (Figure 6).

### RUNX1-205 is much more stable than RUNX1b

**Figure 7:**
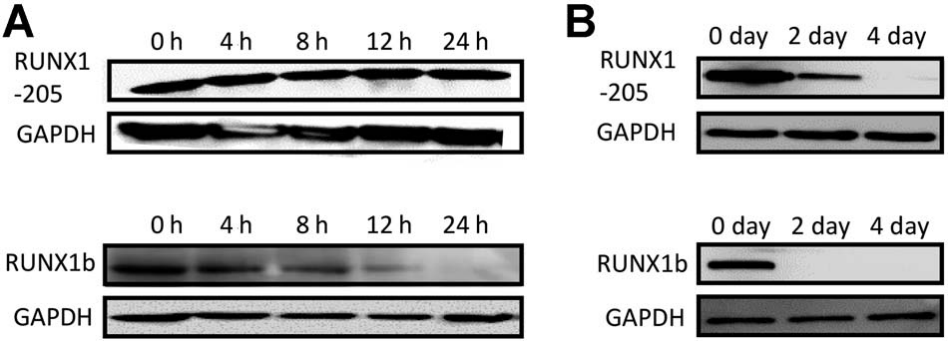
Protein stability of human *RUNX1b* and *RUNX1-205*. Protein samples were prepared from 293T cells with inducible *RUNX1b* or *RUNX1-205* expression at certain time points after DOX was removed. RUNX1b and RUNX1-205 were detected by western blotting within one day (at 0, 4, 8, 12, and 24 h; **A**) or longer time (0, 2, and 4 days; **B**). GAPDH served as a loading control.

Western blot analysis demonstrated that RUNX1-205 was relatively stable after removal of DOX and remained high protein level for one day, whereas RUNX1b gradually disappeared (Figure 7A). Further detection showed that RUNX1-205 was still detectable at D4, whereas RUNX1b could not be detected at D2. The protein half-life of RUNX1-205 is significantly longer than that of RUNX1b (Figure 7B).

### mRNA expression profiles of RUNX1-205 and RUNX1b differ in co-cultured H1 hESCs

**Figure 8:**
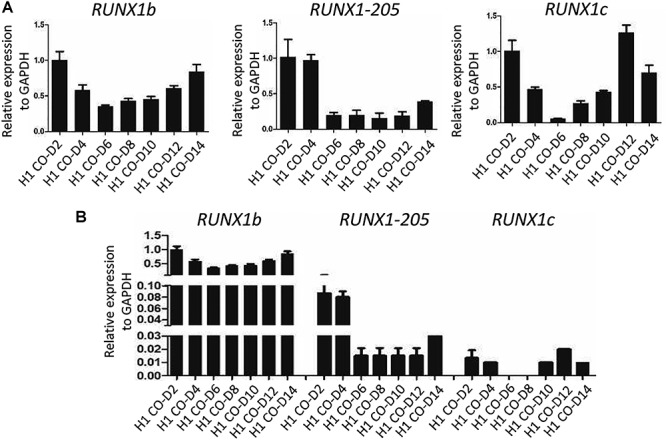
Gene expression profiling analysis of *RUNX1* isoforms during hematopoiesis. Co-cultured H1 hESCs were collected on D2, D4, D6, D8, D10, D12, or D14. (**A**) Relative mRNA expression of *RUNX1b*, *RUNX1-205*, and *RUNX1c* was determined by qRT-PCR analysis. (**B**) The expression levels of these *RUNX1* isoforms were compared. *GAPDH* was used as an internal control.

Although *RUNX1-205* had a similar effect on hematopoiesis as *RUNX1b* (Figures 2–4), qRT-PCR analysis of H1 hESCs co-cultured with AGM-S3 cells revealed that their mRNA expression profiles differed. mRNA expression of *RUNX1-205* was high at early stage (D2–D4), low at late stage (after D4), and upregulated at D14. By contrast, mRNA expression of *RUNX1b* was high at early stage (D2–D4), lowest at D6, and gradually increased at late stage (after D8). mRNA expression of *RUNX1c* was similar to that of *RUNX1b* but exhibited greater fluctuation (Figure 8). Notably, mRNA expression of *RUNX1-205* was much lower than that of *RUNX1b* even at early stage, but was significantly higher than that of *RUNX1c* (Figure 8).

## Discussion

*RUNX1* is essential for human definitive hematopoiesis (Okuda et al., 1996; Chen et al., 2009) and leukemia (Nucifora and Rowley, 1995; Lam and Zhang, 2012). The *RUNX1a/b/c* splice variants of human *RUNX1* have been well studied (Miyoshi et al., 1995; Lacaud et al., 2002; Lorsbach et al., 2004; Brady et al., 2013; Ran et al., 2013; Real et al., 2013; Navarro-Montero et al., 2017). However, the functions of other splice variants, such as *RUNX1-205*, are unclear. High-throughput transcriptome sequencing revealed that *RUNX1-205* homologs are highly conserved in higher vertebrates, including nonhuman primates, mammals, birds, amphibians, reptiles, and fishes (Rennert et al., 2003). Thus, *RUNX1-205* is an ancient splice variant that has been highly conserved during evolution and might play a key role in physiological processes, including hematopoiesis. *RUNX1-205* lacks exon 6, but retains an intact Runt domain and Runx inhibition domain, suggesting that RUNX1-205 and RUNX1b proteins have similar functions. *Runx1-202* in mice is highly homologous to *RUNX1-205* (Komeno et al., 2014). However, the role of *RUNX1-205* in human hematopoiesis and how the absence of exon 6 influences its function must be further explored because hematopoiesis differs between humans and mice (Thomson et al., 1998; Bertrand et al., 2005; Iwasaki and Akashi, 2007).

The function of *RUNX1-205* during hematopoiesis was elucidated using *RUNX1-205*/hESCs and compared with that of *RUNX1b*, which was previously described (Chen et al., 2017). Induction of *RUNX1-205* overexpression at early stage (especially from D0) did not block induction of mesoderm, but blocked mesoderm–hemogenesis transition and the production of CD34^+^CD43^−^ and its progenitor CD34^+^KDR^+^ cells (especially CD34^high^CD43^−^ and CD34^high^KDR^+^ subpopulations) at early stage (Figure 5), which led to the loss of CD34^+^CD43^+^ and CD34^+^CD45^+^ populations at late stage. These effects were attenuated or even abolished by *RUNX1-205* induction at late stage (D6) according to examination on co-culture cells or further hematopoiesis culture, which even indicated the promotion of hematopoietic cells in some degree (Figures 2 and 6). Hematopoietic colony-forming assay confirmed that the hematopoietic potential was blocked by *RUNX1-205* overexpression at early stage, but not affected or even mildly promoted by *RUNX1-205* induction from D6 or later (Figure 4). qRT-PCR analysis demonstrated that various hematopoiesis-related genes were downregulated at D4, which might be related to such blockage effects. A similar effect was observed during EB formation. In general, the earlier *RUNX1-205* overexpression was induced, the inhibitory effect on hematopoiesis was more severe in both co-cultures with AGM-S3 cells and during EB formation. This indicates that induction of *RUNX1-205* overexpression blocks mesoderm−hemogenesis transition, but not later stages, similar to the effect of *RUNX1b*. However, *RUNX1-205* showed a promotion effect during the late stage of hematopoiesis, indicating that compared with *RUNX1b*, it may play different roles at different stages of mesoderm induction and hematopoiesis.

The protein stabilities of RUNX1-205 and RUNX1b in inducible 293T cells were investigated by western blotting. RUNX1-205 was much more stable than RUNX1b. This difference may be due to the absence of exon 6 in *RUNX1-205*. RUNX1b contains nine lysine residues, while RUNX1-205 lacks two of these residues (K182 and K188 in RUNX1b) due to deletion of exon 6. This may mean that RUNX1b is more readily targeted for degradation via the ubiquitin–proteasome pathway, which would explain its lower stability (Tsuji and Noda, 2000; Huang et al., 2001; Biggs et al., 2006). In addition, exon 6 of *RUNX1* is important for methylation of arginine residues. The R206 and R210 residues in the RTAMR region of exon 6 can be methylated by PRMT1, and this prevents the interaction between RUNX1 and SIN3A (Zhao et al., 2008; L'Abbate et al., 2015). By contrast, *RUNX1-205* lacks such interactions. The difference in posttranslational modifications due to the absence of exon 6 in *RUNX1-205* may contribute to the difference in protein stability between RUNX1-205 and RUNX1b. A possible hypothesis is that the alternative splicing mechanism could change the ratio between transcripts of *RUNX1b* and *RUNX1-205* so as to control the ratio of two proteins and their average protein life-time, which might be important for hematopoiesis in early stage.

qRT-PCR analysis revealed that the mRNA expression profiles of *RUNX1-205* and *RUNX1b* differed during hematopoiesis. mRNA expression of *RUNX1-205* was higher at early stage (D2–D4) than at late stage (after D4), suggesting that this isoform mainly functions during mesoderm induction. By contrast, mRNA expression of *RUNX1b* was high at both early (D2–D4) and late (after D8) stages, indicating that this isoform plays important roles throughout hematopoiesis. mRNA expression of *RUNX1-205* was much lower than that of *RUNX1b* even at early stage but was significantly higher than that of *RUNX1*c. This indicates that *RUNX1-205* ought to be an important isoform related to hematopoiesis. Expression of *RUNX1-205* and *RUNX1b* in co-cultures with AGM-S3 cells was high at early stage during generation of mesoderm, indicating that both isoforms are important at this stage, and decreased at the mesoderm−hemogenesis transition. Therefore, the ectopic expression of them at this point might block the transition from mesoderm to hematopoiesis. *RUNX1b* was also important at late stage of hematopoiesis, and consequently its expression gradually increased after D8. However, expression of *RUNX1-205* remained low after D4 and was not upregulated until D14, which indicates that this isoform has a distinct role in comparison with *RUNX1b* at late stage.

Though *RUNX1-205* showed a similar function to *RUNX1b* during hESC differentiation to hematopoiesis, detailed functions of *RUNX1-205* in normal and diseased models still need to be further explored.

## Materials and methods

### Genome structure analysis of RUNX1

Genome sequences, including those of *Homo sapiens* chromosome 21 and *Mus musculus* chromosome 16, were collected from GenBank. The following cDNA sequences were obtained using BLAST searches: chimpanzee NC_027889.1 (primate), house mouse NC_000082.6, zalophus NW_020884868.1, rhinoceros NW_004454184.1 (other mammalian), emu NW_020453926.1 (bird), crocodile NW_017728906.1 (reptile), African frog NC_030727.1 (amphibian), shark NW_018033230.1 (Chondrichthyes, fish), and spearfish NW_005819150.1 (Osteichthyes, fish). Alignments were performed at https://www.genome.jp/tools-bin/clustalw and formatted at https://embnet.vital-it.ch/software/BOX_form.html.

### Construction of a hESC line with inducible RUNX1-205 overexpression

The coding region sequence of *RUNX1-205* was synthesized, cloned into the PUC57 vector, excised using *Sna*BI/*Eco*RI, and inserted between the *Swa*I/*Eco*RI sites of PB-Tet-on-OE to yield the *PiggyBac-*based Tet-on inducible expression vector PB-Tet-on-GFP-T2A-h*RUNX1-205*. H1 hESCs (provided by Prof. Tao Cheng) were transfected with this vector using Lipofectamine 3000 (Invitrogen), selected by treatment with 1 μg/ml puromycin, and passaged using ReleSR (Stem Cell) to establish a hESC line with inducible *RUNX1-205* overexpression (called *RUNX1-205*/hESCs). Induction of *RUNX1-205* overexpression was confirmed by qRT-PCR and western blot analyses. Pluripotency was confirmed by western blot analysis of OCT4, SOX2, and NANOG.

### Co-culture of hESCs and AGM-S3 cells

This study was approved by the institutional ethics committee of the Institute of Blood Transfusion, Chinese Academy of Medical Sciences and Peking Union Medical College (CAMS & PUMC). H1 hESCs were induced to undergo hematopoietic differentiation by co-culture with the mouse stromal cell-derived line AGM-S3 as reported previously (Thomson et al., 1998; Chen et al., 2017). Briefly, undifferentiated *RUNX1b*/hESCs and *RUNX1-205*/hESCs were dissected into small squares containing 0.5 × 10^3^–1 × 10^3^ cells, plated onto irradiated AGM-S3 cells in human pluripotent stem cell-maintaining medium (Chen et al., 2017), cultured for 3 days at 37°C in 5% CO_2_, and then switched to hematopoiesis-inducing medium (defined as Day 0, D0; Chen et al., 2017). The culture medium was changed every day. Cells co-cultured for various numbers of days were dissociated using 0.05%–0.25% trypsin/EDTA (Invitrogen) for further analysis.

### EB formation

*RUNX1b*/hESCs and *RUNX1-205*/hESCs cultured in mTeSR1 were dissociated by treatment with 2 mg/ml dispase (Stem Cells), plated into 24-well ultra-low attachment plates (Corning), and grown in mTeSR1 containing 20 ng/ml bone morphogenetic protein 4 (BMP4) for 24 h (defined as EB Day 0, EB-D0). Thereafter, EBs were dispersed by pipetting and switched to mTeSR1 containing 20 ng/ml BMP4, 100 ng/ml stem cell factor (SCF), and 100 ng/ml Fms-related tyrosine kinase 3 ligand (FL) for 24 h (EB-D1). EBs were switched to EB culture media (IMDM containing 20 ng/ml BMP4, 100 ng/ml SCF, 100 ng/ml FL, 20% fetal bovine serum (FBS), 1 mM L-glutamine, 0.1 mM β-mercaptoethanol, and 1% nonessential amino acids) at EB-D2. EBs were treated with 1 μg/ml DOX from EB-D0, EB-D2, EB-D4, or EB-D6, collected at EB-D10, and digested with 0.05% trypsin for further analysis.

### FACS analysis of inducible expression of RUNX1 isoforms

Co-cultures of *RUNX1b*/hESCs or *RUNX1-205*/hESCs and AGM-S3 cells were treated with 1 μg/ml DOX (referred to as DOX-induced) from D0, D2, D4, D6, D8, or D10 and then dissociated at D4, D8, or D14 with 0.05%–0.25% trypsin/EDTA, which was inactivated by cold Dulbecco’s phosphate-buffered saline (D-PBS). Cells were pelleted, resuspended in 1 ml sorting medium (D-PBS containing 2% FBS), passed through a 70-μm membrane filter, blocked with rabbit serum on ice for 20 min, labeled with antibodies against CD34/KDR (at D4), CD34/CD43 (at D8), or CD34/CD45 (at D14), and analyzed by FACS (Okumura et al., 1992; Vodyanik et al., 2006). FACS was performed using a flow cytometry system (Canto II, BD) and data were processed using FlowJo V10.

### Protein stability assay

293T cells (ATCC) were transfected with *RUNX1b* or *RUNX1-205* inducible vectors, selected using 1 μg/ml puromycin, and treated with 1 μg/ml DOX for 48 h. DOX was then removed (defined as 0 h). Protein samples were collected at 0 (0 day), 4, 8, 12, 24, 48 (2 days), and 96 h (4 days) and analyzed by western blotting.

### qRT-PCR analysis

Total RNA was extracted from 0.5 × 10^6^–1 × 10^6^ cells using 1 ml TRIzol (Life Technologies) and purified according to the manufacturer’s manual. Complementary DNA was synthesized using a reverse transcription kit (Bio-Rad). Each 20 μl reaction contained 4 μl 5× Mixture, 1 μl reverse transcriptase, 1 μg total RNA, and nuclease-free water to 20 μl. The conditions used for reverse transcription were as follows: 25°C for 5 min, 42°C for 30 min, 85°C for 5 min, and storage at 4°C. qPCR was performed using Fast Start Universal SYBR Green Master (Roche) on a CFX96™ real-time system (Bio-Rad). Each 15 μl reaction contained 7.5 μl 2× Mixture, 0.4 μl each primer (10 μM), 4.7 μl H_2_O, and 2 μl complementary DNA. The conditions were as follows: denaturation at 10 min at 95°C, followed by 45 cycles of 95°C for 15 sec, 58°C for 30 sec, and 72°C for 30 sec. Glyceraldehyde 3-phosphate dehydrogenase (*GAPDH*) served as an internal control. The primers are listed in Supplementary Table S1.

### Hematopoietic colony-forming assay

The hematopoietic colony-forming assay of co-cultured cells was performed as described previously (Ma et al., 2008; Li et al., 2014). At D14, co-cultured cells were dissociated by 0.25% trypsin/EDTA. Thereafter, 5 × 10^4^ co-cultured cells were added to 80% MethoCult H4230 (Stem Cell) containing 100 ng/ml SCF, 100 ng/ml interleukin-6 (IL-6), 10 ng/ml interleukin-3 (IL-3), 10 ng/ml FL, 10 ng/ml thrombopoietin (TPO), 10 ng/ml granulocyte-macrophage colony-stimulating factor (GM-CSF), and 4 units/ml erythropoietin (EPO) and then incubated in 5% CO_2_ at 37°C for 14 days. The number of CFU-E colonies was calculated at 7–10 days, while the numbers of BFU-E, CFU-Mix, and CFU-GM colonies were calculated at 12–14 days as previously described (Mao et al., 2016).

### MGG staining

Cells in BFU-E colonies were harvested and spun onto glass slides using a Cytospin 4 Cytocentrifuge (Thermo Fisher Scientific). For morphological observation, cells were stained with MGG solution (MERCK) and then imaged using an Olympus BX53 microscope equipped with an oil objective.

### Cell sorting and endothelium culture

Noninduced or induced *RUNX1-205*/hESC co-cultures at D2 were dissociated with 0.05% trypsin solution and stained with anti-KDR antibody. KDR^+^ cells were sorted using a BD FACSJazz™ Cell Sorter, and their purity was confirmed by FACS. About 5 × 10^3^ sorted cells were re-plated in 24-well plate on irradiated AGM-S3 for 10 days with or without DOX induction, refreshed with hematopoiesis-inducing medium every other day, and finally analyzed by FACS analysis.

### Assay of full-lineage hematopoietic differentiation

*RUNX1-205*/hESC co-cultures at D6 were dissociated with 0.05% trypsin solution and stained with anti-CD34/KDR antibody. Then CD34^+^KDR^−^ cells were sorted, and ~5 × 10^3^ cells were re-plated in 48-well plate with full-lineage hematopoietic differentiation (FLHD) medium (IMDM containing 10% FBS, 100 ng/ml SCF, 100 ng/ml IL-6, 10 ng/ml IL-3, 10 ng/ml FL, 10 ng/ml TPO, and 4 IU/ml EPO) for 5 days treated with or without DOX, refreshed with media every day, and finally analyzed by FACS analysis.

### Statistical analysis

All experimental data were described as mean ± SD. Statistical significance was evaluated using the Student’s *t*-test. *P* < 0.05 was considered significant. Data were analyzed using FlowJo V10 and GraphPad Prism 5 software.

## Supplementary Material

Supplementary_material_mjaa019
