# Supplementary material for: RUNX1-205, a novel splice variant of the human RUNX1 gene, has blockage effect on mesoderm–hemogenesis transition and promotion effect during the late stage of hematopoiesis
Source: J Mol Cell Biol. 2020 Apr 21;12(5):386–96. doi: 10.1093/jmcb/mjaa019 (PMC7288743; doi:10.1093/jmcb/mjaa019)
Supplement: Supplementary_material_mjaa019 [file supplementary_material_mjaa019.pdf]

Supplementary material

RUNX1-205, a novel splice variant of the human *RUNX1* gene, has blockage effect on mesoderm–hemogenesis transition and promotion effect during the late stage of hematopoiesis

Wencui Sun[1,†], Jiahui Zeng[1,†], Jing Chang[1,†], Yuan Xue[1], Yonggang Zhang[1], Xu Pan[1], Ya Zhou[1], Mowen Lai[1], Guohui Bian[1], Qiongxiu Zhou[1], Jiaxing Liu[1], Bo Chen[1,*], and Feng Ma[1,2,3,*]

[1] Institute of Blood Transfusion, Chinese Academy of Medical Sciences & Peking Union Medical College (CAMS & PUMC), Chengdu 610052, China

[2] State Key Laboratory of Biotherapy, Sichuan University, Chengdu 61006, China

[3] State Key Laboratory of Experimental Hematology, CAMS & PUMC, Tianjin 300020, China

[†]These authors contributed equally to this work.

*Correspondence to: Bo Chen, Tel: +86-61648515, Fax: +86-28-8332125, E-mail: 18980999910@163.com; Feng Ma, Tel: +86-61648510, Fax: +86-28-8332125, E-mail: mafeng@hotmail.co.jp

Supplemental materials and methods

Western blotting

Cells were lysed in total lysis buffer (12 mM Tris-HCl, pH 8.3, 100 mM NaCl, 1% SDS, 1% DCA, 1% Triton X-100, 2 mM EDTA, 50 μM DTT, and 2 mM PMSF) with shaking on ice for 30 min. After centrifugation (12,000 ×g, 4 ℃, 5 min), the protein concentration of the supernatant was determined by the classic BCA method. Proteins were denatured at 100 ℃ for 5 min and then electrophoresed by denaturing SDS-PAGE (5% separating gel/10% stacking gel) according to the standard protocol of the Bio-Rad Mini-PROTEAN Tetra Cell electrophoresis system. Proteins were transferred to a PVDF membrane using a Bio-Rad Trans-Blot cell in 1 × TBST (10 mM Tris-HCl, pH8.0, 150 mM NaCl, and 0.05% Tween 20). Thereafter, membranes were incubated with anti-SOX2 (diluted 1:1000; Cat.#sc-365823, SANTA CRUZ), anti-OCT3/4 (diluted 1:1000; Cat.#sc-5279, SANTA CRUZ), anti-NANOG (diluted 1:1000; Cat.#sc-293121, SANTA CRUZ), anti-RUNX1 (diluted 1:100; Cat.#BM-2579, BOSTER), and anti-GAPDH (diluted 1:1000; Cat.#KC-5G4, KANG CHEN) mouse monoclonal IgGs in blocking solution (1 × TBST containing 5% non-fat milk powder) overnight at 4 ℃. Membranes were then washed with 1 × TBST, incubated with horseradish peroxidase-conjugated goat anti-mouse IgG H&L (diluted 1:1000; Cat.#ZB-2305, ZSBIO) in blocking solution, washed again with 1 × TBST, and finally developed using an Amersham ECL Prime western blotting detection kit (Cat.#RPN2232, GE Healthcare). Signals were quantified using an ImageQuant LAS4000 mini system. Molecular weights were calculated by reference to the pre-stained protein ladder (Cat. # 26626, PageRuler).

Supplementary Table S1 Primers used for qRT-PCR analysis.

Gene	Forward primer (5′→3′)	Reverse primer (5′→3′)
GAPDH	GTCTCCTCTGACTTCAACAGCG	ACCACCCTGTTGCTGTAGCCAA
KDR	GGAACCTCACTATCCGCAGAGT	CCAAGTTCGTCTTTTCCTGGGC
RUNX1b	TCTGCAGAACTTTCCAGTCG	GTCGGGGAGTAGGTGAAGG
RUNX1c	CCTTCGTACCCACAGTGCTT	CAACGCCTCGCTCATCTT
RUNX1-205	CCCCGAGAACCTCGAAATAC	CGTCGGGGAGTAGGTGAAG
GATA1	CACGACACTGTGGCGGAGAAAT	TTCCAGATGCCTTGCGGTTTCG
GATA2	CAGCAAGGCTCGTTCCTGTTCA	ATGAGTGGTCGGTTCTGCCCAT
GATA3	GCGAGACAGAGCGAGCAA	ACTGGGTACGGCAGAATAAAA
PU.1	GACACGGATCTATACCAACGCC	CCGTGAAGTTGTTCTCGGCGAA
c-KIT	CACCGAAGGAGGCACTTACACA	TGCCATTCACGAGCCTGTCGTA
vWF	CCTTGAATCCCAGTGACCCTGA	GGTTCCGAGATGTCCTCCACAT
CD34	AACATCTCCCACTAAACCCTA	TCTTAAACTCCGCACAGCTG

Supplementary figures

Supplementary Figure S1 (A) Exon-intron structure of human *RUNX1b* and *RUNX1-205* and mouse *Runx1b* and *Runx1-202*. Boxes represent exons. White represents untranslated regions, and black represents coding sequences. P1, distal promoter; P2, proximal promoter. (B) Alignment of human *RUNX1* isoforms and mouse *Runx1* isoforms. (C) Conservation analysis of *RUNX1-205* homologs. Red boxes denote the Runt domain and Runx inhibition domains of *RUNX1*.

Supplementary Figure S2 Construction of (A) PB-Tet-on-GFP-T2A-h*RUNX1b* and (B) PB-Tet-on-GFP-T2A-h*RUNX1-205*.

Supplementary Figure S3 Co-cultured *RUNX1b*/hESCs or *RUNX1-205*/hESCs were treated with DOX from D0, D2, D4, or D6 and analyzed by FACS at D4, D8, or D14 using antibodies against CD34/KDR, CD34/CD43, or CD34/CD45. The (A) CD34+KDR- and CD34-KDR+ populations at D4, (B) CD34+CD43-, CD34-CD43+, and CD34+CD43+ populations at D8, and (C) CD34+CD45-, CD34-CD45+, and CD34+CD45+ populations at D14 were compared between non-induced co-cultures and the GFP+ fraction of co-cultures treated with DOX from D0, D2, D4, or D6.

Figure S1

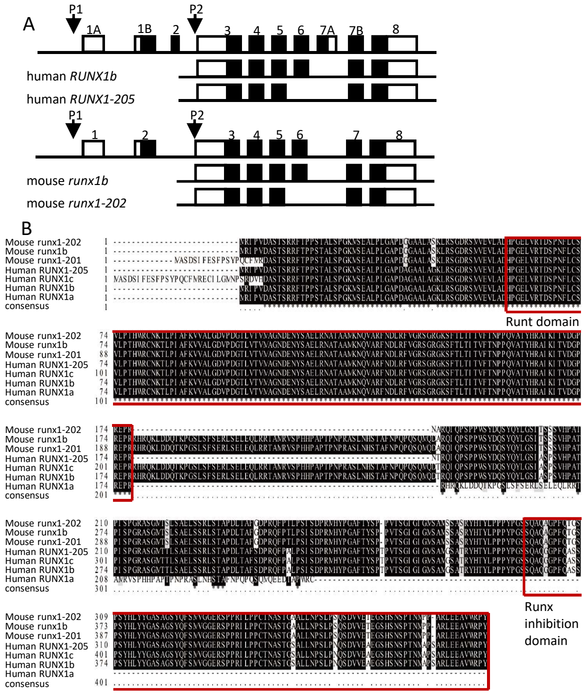
A
P1
P2
1A
1B
2
3
4
5
6
7A
7B
8
human RUNX1b
human RUNX1-205
1
7
mouse runx1b
mouse runx1-202
B
Mouse runx1-202
Mouse runx1b
Mouse runx1-201
Human RUNX1-205
Human RUNX1c
Human RUNX1b
Human RUNX1a
consensus
Runt domain
Runx inhibition domain

C

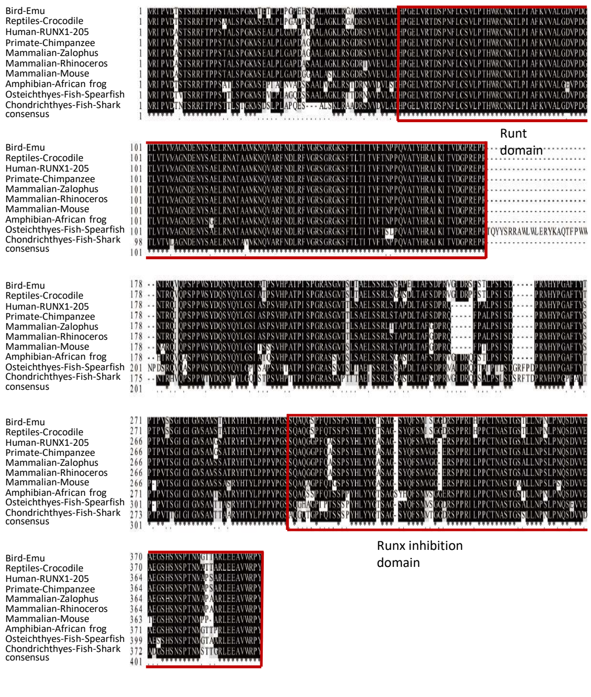
Bird-Emu
Reptiles-Crocodile
Human-RUNX1-205
Primate-Chimpanzee
Mammalian-Zalophus
Mammalian-Rhinoceros
Mammalian-Mouse
Amphibian-African frog
Osteichthyes-Fish-Spearfish
Chondrichthyes-Fish-Shark
consensus
Runt domain
Runx inhibition domain

Figure S2

A

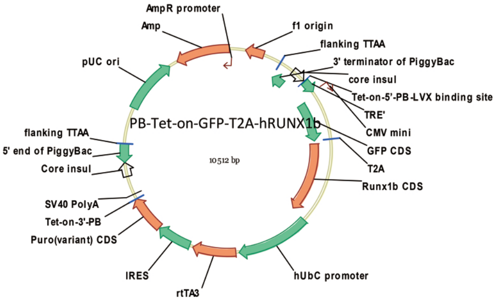
AmpR promoter
Amp
f1 origin
flanking TTAA
3' terminator of PiggyBac
core insul
Tet-on-5'-PB-LVX binding site
TRE'
CMV mini
GFP CDS
T2A
Runx1b CDS
pUC ori
PB-Tet-on-GFP-T2A-hRUNX1b
10512 bp
flanking TTAA
5' end of PiggyBac
Core insul
SV40 PolyA
Tet-on-3'-PB
Puro(variant) CDS
IRES
rtTA3
hUbC promoter

B

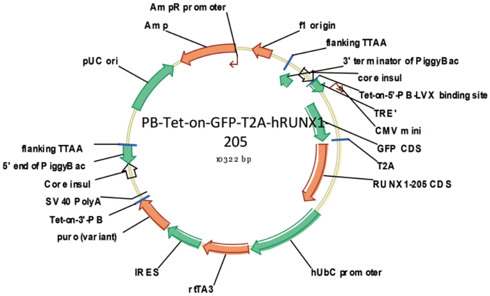
AmpR promoter
Amp
f1 origin
flanking TTAA
3' terminator of PiggyBac
core insul
Tet-on-5'-PB-LVX binding site
TRE'
CMV mini
GFP CDS
T2A
RUNX1-205 CDS
pUC ori
PB-Tet-on-GFP-T2A-hRUNX1
205
10322 bp
flanking TTAA
5' end of PiggyBac
Core insul
SV40 PolyA
Tet-on-3'-PB
puro (variant)
IRES
rtTA3
hUbC promoter

Figure S3

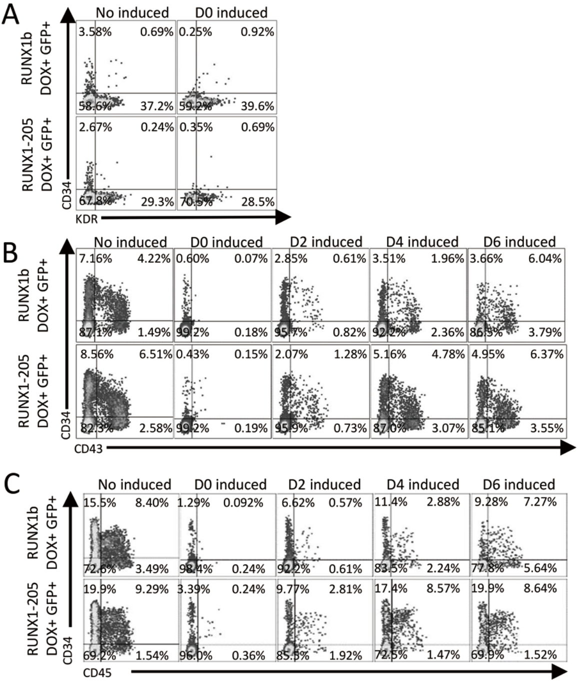

A
No induced
D0 induced
RUNX1b DOX+ GFP+
3.58%
0.69%
0.25%
0.92%
58.6%
37.2%
59.2%
39.6%
RUNX1-205 DOX+ GFP+
2.67%
0.24%
0.35%
0.69%
67.8%
29.3%
70.5%
28.5%
CD34
KDR
B
No induced
D0 induced
D2 induced
D4 induced
D6 induced
RUNX1b DOX+ GFP+
7.16%
4.22%
0.60%
0.07%
2.85%
0.61%
3.51%
1.96%
3.66%
6.04%
87.1%
1.49%
99.2%
0.18%
95.7%
0.82%
92.2%
2.36%
86.5%
3.79%
RUNX1-205 DOX+ GFP+
8.56%
6.51%
0.43%
0.15%
2.07%
1.28%
5.16%
4.78%
4.95%
6.37%
82.3%
2.58%
99.2%
0.19%
95.9%
0.73%
87.0%
3.07%
85.1%
3.55%
CD34
CD43
C
No induced
D0 induced
D2 induced
D4 induced
D6 induced
RUNX1b DOX+ GFP+
15.5%
8.40%
1.29%
0.092%
6.62%
0.57%
11.4%
2.88%
9.28%
7.27%
72.6%
3.49%
98.4%
0.24%
92.2%
0.61%
83.5%
2.24%
77.8%
5.64%
RUNX1-205 DOX+ GFP+
19.9%
9.29%
3.39%
0.24%
9.77%
2.81%
17.4%
8.57%
19.9%
8.64%
69.2%
1.54%
96.0%
0.36%
85.5%
1.92%
72.5%
1.47%
69.9%
1.52%
CD34
CD45
